# State-dependent domicile leaving rates in *Anopheles gambiae*

**DOI:** 10.1186/s12936-017-2166-4

**Published:** 2018-01-12

**Authors:** Simon P. W. Zappia, Alex M. Chubaty, Bernard D. Roitberg

**Affiliations:** 1 Evolutionary and Behavioural Ecology Research Group, Department of Biological Sciences, Simon Fraser University, Burnaby, BC Canada; 2 Natural Resources Canada, Canadian Forest Service, Victoria, BC Canada; 3 Faculté de foresterie, de géographie et de géomatique, Département des sciences du bois et de la forêt, Université Laval, Québec, Canada

**Keywords:** Energy, Time, State-dependent behaviour, Floral cues, Domicile emigration

## Abstract

**Background:**

Transmission of *Plasmodium* greatly depends on the foraging behaviour of its mosquito vector (*Anopheles* spp.). The accessibility of blood hosts and availability of plant sugar (i.e., nectar) sources, together with mosquito energy state, have been shown to modulate blood feeding (and thus biting rates) of anopheline mosquitoes. In this study, the influence of mosquito starvation status and availability of nectar on the decision of female *Anopheles gambiae* mosquitoes to leave a bed net-protected blood host was examined.

**Methods:**

Two small-scale mesocosm experiments were conducted using female mosquitoes starved for 0, 24 or 48 h, that were released inside a specially constructed hut with mesh-sealed exits and containing a bed net-protected human volunteer. Floral cues were positioned on one side of the hut or the other. Several biologically plausible exponential decay models were developed that characterized the emigration rates of mosquitoes from the huts. These varied from simple random loss to leaving rates dependent upon energy state and time. These model fits were evaluated by examining their fitted parameter estimates and comparing Akaike information criterion.

**Results:**

Starved mosquitoes left domiciles at a higher rate than recently fed individuals however, there was no difference between 1- and 2-day-starved mosquitoes. There was also no effect of floral cue placement. The best fitting emigration model was one based on both mosquito energy state and time whereas the worst fitting model was one based on the assumption of constant leaving rates, independent of time and energy state.

**Conclusions:**

The results confirm that mosquito-leaving behaviour is energy-state dependent, and provide some of the first evidence of state-dependent domicile emigration in *An. gambiae*, which may play a role in malarial transmission dynamics. Employment of simple, first-principle, mechanistic models can be very useful to our understanding of why and how mosquitoes leave domiciles.

**Electronic supplementary material:**

The online version of this article (10.1186/s12936-017-2166-4) contains supplementary material, which is available to authorized users.

## Background

Malaria dynamics may depend greatly on the distribution of hosts, vectors, the ease of movement between human habitations (where *Anopheles* mosquitoes forage for blood), and on the availability and distribution of plant sugars (i.e., nectars) [1, 2]. Blood meals provide protein for mosquito egg maturation and nectars fuel flight and provide for somatic maintenance [3]. In general, blood and sugar sources are spatially separate and require separate foraging bouts [4], thus the presence or absence of readily available sugar sources may shed light on the mechanisms that might modify the distribution and spatial heterogeneity of anopheline mosquitoes.

It is now recognized that sugar (generally nectar) is critical to survival of *Anopheles* mosquitoes in nature, for both males and females [e.g., 5]. Individuals may feed at nectaries, extra-floral nectaries or other sources of carbohydrates (e.g., rotting fruit) [6]. Mosquitoes likely locate sugar sources by olfactory [7] and visual means [8] and may discriminate between different sugar sources [9]. Of course, many of these nectar sources are located outside of domiciles, thus, as noted above, sugar sources may impact the distribution of resource-seeking mosquitoes in space and time.

The goal of this research is to employ controlled experiments to study an open system where there is free movement within and among human domiciles to pinpoint what internal and external factors affect mosquito movement from domiciles. Recent theoretical studies suggest that mosquito populations are susceptible to sugar-based interventions outside of domiciles, including toxic sugar baits [10].

Previous modelling and laboratory work [e.g., 2, 11, 12] suggest that when mosquitoes enter a domicile, they face the (adaptive) choice of trying to obtain a blood meal or, if the host is unobtainable, leave the habitation and pursue either a different host in another domicile or access sugar resources from plants that may be present some distance away. Either choice might be dictated by, among other things, a combination of the ease of feeding on a blood host [13], the danger associated with feeding [14], the mosquito’s energy status [15], and the perceived likelihood of successfully encountering an alternative energy source within the time remaining for host search [16]. This is in keeping with state dependent foraging theory as detailed for a broad range of organisms that forage for discrete resources [17]. Further, given the limited body size of a mosquito [18], meals become mutually exclusive. Hence, meal choice is key to the mosquito’s lifetime fitness as sugar energy (which is generally found outdoors) can only be used for flight while blood (mostly found indoors) is employed for both flight and oogenesis. In the laboratory, it was shown that moderately starved mosquitoes will actively abandon protected blood hosts and fly to easily accessible sugar resources [12] whereas in mesocosms, female *Anopheles gambiae* only favoured sugar sources over blood hosts for first meals when their energy reserves were very low (with body size as a proxy for energy state) [19].

Given these premises, analysing emigration rates from human domiciles is important when considering how efficiently a vector-borne disease is transmitted, because biting frequency and mosquito survival can dramatically affect *Plasmodium* transmission [e.g., 20, 21], and are inherently affected by the ease of obtaining blood and sugar, respectively [1]. Recent publications point out the importance of understanding how vectors respond to different environmental parameters when employing integrated intervention tools [e.g., 21].

How a mosquito’s energy status affects its host-seeking behaviour has been investigated in small-scale experiments in which mosquitoes were confronted with an uncooperative [13, 22] or unobtainable [12] blood host under different energy or body-size [19] conditions. These studies have shown that mosquitoes are more persistent at attacking a blood host when starved of sugar and that mosquitoes with either very high or very low energy status are less prone to leaving the immediate vicinity of a blood host even when presented with an obtainable sugar meal option a short distance away. These studies, though useful, have mostly been conducted in small laboratory settings where mosquitoes were observed for a short period of time in very confined environments, where access to both blood and its alternative carbohydrate source was relatively easy (i.e., short flights < 1.5 m). Yet, what happens when spatial proportions mimic reality in a mesocosm system encompassing a life-sized human habitation has yet to be assessed for state-dependent emigration decisions (but see [23] for semi-natural olfactometer tests and the aforementioned mesocosm study with nectar plants [19]).

Adding further complexity to the questions raised above is the mechanism by which mosquitoes locate their sugar (nectar) hosts. Like most herbivorous arthropods, *An. gambiae* individuals likely locate hosts via plant semiochemicals [24]. A number of floral odours have been demonstrated to be attractive to mosquitoes in laboratory settings [7]; however, their attractiveness when immersed in other naturally occurring odour plumes is less clear. Recent work shows that *Anopheles arabiensis* can readily locate fruit-sugar baits that compete within *Ricinus communis* vegetation [25]. However, Stone et al. showed that *An. gambiae* females preferred human blood hosts to nectar bearing *Senna didymobotrya* plants [19].

Two questions arise with regard to the above discussion: (i) under full-scale conditions, is energy state a driver of emigration, similar to the results from our small-scale experiments? and, (ii) under natural conditions where the signal to noise ratio from floral cues could be much diminished, are *An. gambiae* females likely to abandon protected blood host locales in search of sugar sources, as found in the laboratory?

Two experiments were run that utilized similar methodologies. Experiment one, investigated how the energy state of *An. gambiae* affects its emigration rates from a life-sized hut in which an unobtainable human blood host was sheltered. Here, any floral odour was uncontrolled and may have come from considerable distance (tens of metres). Here, any floral odour was uncontrolled and may have come from considerable distance (tens of metres). The second experiment, controlled for presence/absence of easily obtainable, nearby (1 m) floral resources but again, within a matrix of other uncontrolled floral odours that emanated from considerable distance.

## Methods

All work was carried out at the International Centre for Insect Physiology and Ecology (ICIPE), situated on Lake Victoria, at Mbita, Kenya, East Africa time zone (EAT).

### Mosquito colony

These experiments used individual mosquitoes obtained from a laboratory colony of *An. gambiae* Mbita strain. Adults were fed on 5% glucose solution ad libitum from a rolled-up, dye-free paper towel placed in a cylindrical plastic container. Mosquitoes were kept in custom-made metal-framed cages (30 cm × 30 cm × 30 cm) lined with white polyester mesh. These cages were enclosed in a screen-house exposed to natural sunlight and daytime cycle at temperatures that ranged between 30 and 17 °C (day/night, respectively) and relative humidity of 68%. To obtain eggs, adult females were allowed to feed on a human arm for 8–10 min immediately after sunset. Gravid mosquitoes were then allowed to oviposit on moist filter paper 3 days after blood feeding. The eggs were collected and thoroughly washed with distilled water to induce hatching. Once hatched, batches of 200 first instar larvae were placed individually in large plastic pans (45 cm × 15 cm, water depth 3–4 cm) containing filtered lake water and provided with finely ground lyophilized fish flake food (Sera^®^) *ad lib*. Pupae were individually picked and transferred to water-filled transparent plastic cups awaiting eclosion. A total of 3555 females was used. These female mosquitoes had wing length between 2.66 and 3.38 mm (mean 2.97 mm), 5–7 days post-eclosion, not previously blood-fed and presumed mated (copulations were observed in the cages).

### Effect of starvation treatments on leaving rate

#### Experimental set-up

The goal of this experiment was to elucidate how mosquito-leaving rates vary over the course of the evening as mosquitoes exhaust their energy stores and approach end of night. This approach mirrors events in and around domiciles in East Africa.

**Fig. 1:**
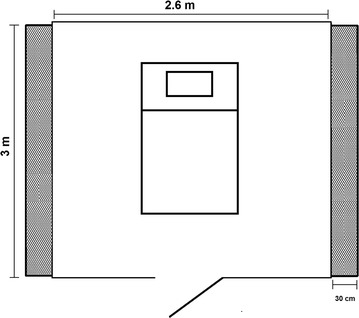
Sketch of the specially constructed experimental hut (height: 2 m; see description in text). Overall surface area of walls, floor, and ceiling was 38 sq m, and the surface area of the traps was 0.15 sq m

Two two identical wooden, rectangular-prism, huts (3 m × 2.6 m × 2 m) were built, each with a small front door, no windows. The walls were made of lightly tanned plywood to make mosquito detection easier. Gaps between the plywood sheets, beam structure and exit traps were sealed using cotton wool. On the left and right walls of the hut, a gap was allowed between the wall and the base of the roof (located 1.5 m from the floor) that extended the full width of the wall for exit trap placement. The exit traps (Fig. 1) were made of stiff metallic wire frame and lined with untreated and unscented fine white polyester mesh (3 m × 0.3 m × 0.2 m). The inner part of the trap had two flaps folded at approximately 45° with the intent of making mosquito re-entry into the hut unlikely. To shelter the two huts from wind and rain, an external structure (11 m × 4.6 m) lined with reed mats was also constructed. The exit traps were comparable to those of Müller et al. [26]; however they differed in that individuals could readily pass through the mesh in this study rather than being caught on glue. The Müller et al. design is likely more efficient in that some mosquitoes from the current experiment were never recovered inside the domicile or in the exit trap, whereas, in the glue-capture design, most exiting individuals were caught.

In each of the huts, one of four human volunteers was chosen to sleep on a mattress placed in the middle of the hut under the protection of an untreated white bed net suspended from the ceiling. Volunteers were selected haphazardly, with replacement, based on their availability, and to ensure that all combinations of treatment and volunteer were used at least 5 times.

In each of the huts, one of four human volunteers was chosen to sleep on a mattress placed in the middle of the hut under the protection of an untreated white bed net suspended from the ceiling.

#### Experimental procedure

To set up the experimental replicates, female mosquitoes (average of 48 females per hut per night) were haphazardly collected using a mouth aspirator, from maintenance cages that had received a randomly assigned treatment of either 0, 24 or 48 h starvation from sugar, where the sugar source was substituted with distilled water. The mosquitoes were then placed in a paper cup approximately 30 min before the beginning of the trial. All experiments started at 20:00 EAT (approximately 30 min past sunset, in total darkness). Females were released from the paper cup at the foot of the bed, in front of the door by the volunteer. Following release, the volunteer remained under the safety of the bed net, taking care to not contact the net with exposed skin. Individual runs were terminated at 23:00, 03:00 or 07:00 EAT (sunrise), at which point, the volunteer would seal the exit traps for later mosquito collection (i.e., any further emigration was halted at each of those time points to give cumulative emigration up to those independent termination points). At 07:00, any mosquitoes still in the hut were gathered using a backpack aspirator, while the ones in the exit traps were captured using a mouth aspirator.

#### Wing measurement

Collected specimens were frozen for several hours, tallied and placed in a drying oven at 45 °C for a minimum of 3 days. Wings were then dissected, photographed and their lengths recorded using the software Analyzing Digital Images, Version 11-2008 [27]. Mosquito wing length serves as a good proxy for female body size [per, 28]. Females that were much larger or much smaller than average (i.e., outliers) were omitted from subsequent analyses.

#### Data analyses

To test for an effect of starvation treatments on leaving rate, the proportions of mosquitoes leaving the hut for each starvation treatment, using a logistic regression. Likewise, the effect of experiment stop time on the proportion of mosquitoes leaving the hut in each starvation treatment was evaluated. All analyses, except those indicated below, were performed using the R statistical language and environment version 3.4.3 [29].

### Floral odour interaction

When investigating floral odour interactions, the same procedure as the previous experiment was followed but also included two plants described as being attractive to mosquitoes for sugar-feeding purposes: *Parthenium hysterophorus* and *Ricinus communis* [30]. A total of four plants—two of each type—were grown in pots and hung on the outside edge of a randomly selected trap in alternating order such that their odour could permeate the hut without interfering with mosquito movement. Female mosquitoes subjected to two starvation levels (either 0 or 24 h).

A Chi squared test (based upon logistic regression) was used to assess differences in mosquito emigration between a trap baited with attractive plants and a control.

### Curve fitting and model selection

Five different models (i.e., hypotheses) characterizing the leaving behaviour of mosquito populations were fit to the experimental data using non-linear least squares regression. Each of these hut-leaving models is based on a simple exponential decay model describing the proportion of mosquitoes remaining in the hut (*p*) over time (*t*). These model fits were evaluated by examining their fitted parameter estimates, comparing Akaike information criterion (AIC) scores, and by visual examination of the residuals.

#### Model 0: background leaving rate independent of energy state

Individuals actively leave the hut throughout the evening at some constant rate, but this rate (*a*) is independent of energy state.

$$p_{0} = e^{ - at}$$

#### Model 1: background leaving rate based on energy state (treatment)

Individuals accidentally leave the main area when they fall through the trap mesh. This rate (*f*_*x*_) is dependent upon the ratio of trap surface area (0.15 m^2^) to domicile surface area (38 m^2^), as well as mosquito energy state (*x*).

$$p_{1} = e^{{ - f_{x} t}}$$

#### Model 2: leaving rate based on energy state (treatment)

Individuals actively leave the hut throughout the evening at some constant rate (*a*_*x*_). This rate is a function of the energy state manipulation, and this equation is mathematically identical to Model 1.

$$p_{2} = e^{{ - a_{x} t}}$$

#### Model 3: some individuals leave immediately, others leave gradually based on energy state

Same as Model 2 above, but some proportion of individuals escape the hut immediately (*p*_*l*_), and the remainder leave at rate *a*_*x*_. This model was constrained to parameter estimates that resulted in a decline in *p*_*in*_ over time (i.e., *a*_*x*_ ≥ 0), to match the experimental scenario where mosquitoes can only leave the hut.

$$p_{3} = (1 - p_{l} )e^{{ - a_{x} t}}$$

#### Model 4: leaving rate dependent on both energy state and time

Like Model 2, individuals use up energy over time, leaving the hut at some rate, *a*_*x,t*_, that is dependent upon both *x* and *t*.

$$p_{4} = e^{{ - a_{x,t} t}}$$

## Results

### Effect of starvation treatments on leaving rate

Significant differences between starved and unstarved mosquitoes in the proportion of individuals leaving the hut (Table 1); however, there was not a significant difference between mosquitoes starved for 24 h versus 48 h (χ^2^ = 1.3; df = 1; p = 0.25). There was no effect of experiment end time on the proportion of mosquitoes leaving the hut across the three starvation treatments (p = 0.1; see Additional file 1).

**Table 1:** Evaluation of mosquito leaving rates based on starvation treatment using logistic regression

Parameter	Estimate	Std. Error	z value	Pr(>|z|)
Intercept	− 2.07555804	0.093405266	− 22.2209961	< 2.2e−16
Treatment24h	0.66078421	0.119368753	5.5356548	3.1006777e−08
Treatment48h	0.53950268	0.120236196	4.4870238	7.2225000e−06
			Null df = 71	Residual df = 69

**Table 2:** Summary of model fits

Model	Parameter	Treatment	Estimate	Std. error	t value	Pr(>|t|)
0	*a*_*0*_	NA	0.021443287	0.001895014	11.315637	1.493977e−17
2	*a*_*x*_ *1*	0 h	0.013086139	0.002758499	4.743934	1.093659e−05
	*a*_*x*_ *2*	24 h	0.027286511	0.003260572	8.368627	4.232912e−12
	*a*_*x*_ *3*	48 h	0.025137449	0.003196740	7.863464	3.552471e−11
3a	*a*_*x*_ *1*	0 h	0.000000000	0.003715630	0.000000	1.000000e+00
	*a*_*x*_ *2*	24 h	0.011569714	0.004062655	2.847821	5.816893e−03
	*a*_*x*_ *3*	48 h	0.009578528	0.004021740	2.381687	2.003796e−02
	*p*_*l*_	NA	0.119399364	0.022798384	5.237185	1.725421e−06
4	*a*_*x,t*_ *1*	0 h_11	0.009092421	0.002572912	3.533903	7.731737e−04
	*a*_*x,t*_ *2*	0 h_3	0.040767387	0.010938229	3.727056	4.167879e−04
	*a*_*x,t*_ *3*	0h_7	0.018668330	0.004421931	4.221760	7.935037e−05
	*a*_*x,t*_ *4*	24 h_11	0.023808634	0.003208514	7.420454	3.685148e−10
	*a*_*x,t*_ *5*	24 h_3	0.074331434	0.011315691	6.568882	1.120628e−08
	*a*_*x,t*_ *6*	24 h_7	0.024330330	0.004600709	5.288387	1.648487e−06
	*a*_*x,t*_ *7*	48 h_11	0.022587395	0.003165701	7.135038	1.161947e−09
	*a*_*x,t*_ *8*	48 h_3	0.058629404	0.010795013	5.431157	9.584620e−07
	*a*_*x,t*_ *9*	48 h_7	0.023216988	0.004564993	5.085876	3.528368e−06

### Floral odour interaction

No significant differences were detected when comparing the proportion of mosquitoes escaping in plant-baited traps *versus* control traps in either starvation treatments (p = 0.18).

### Curve fitting and model selection

**Fig. 2:**
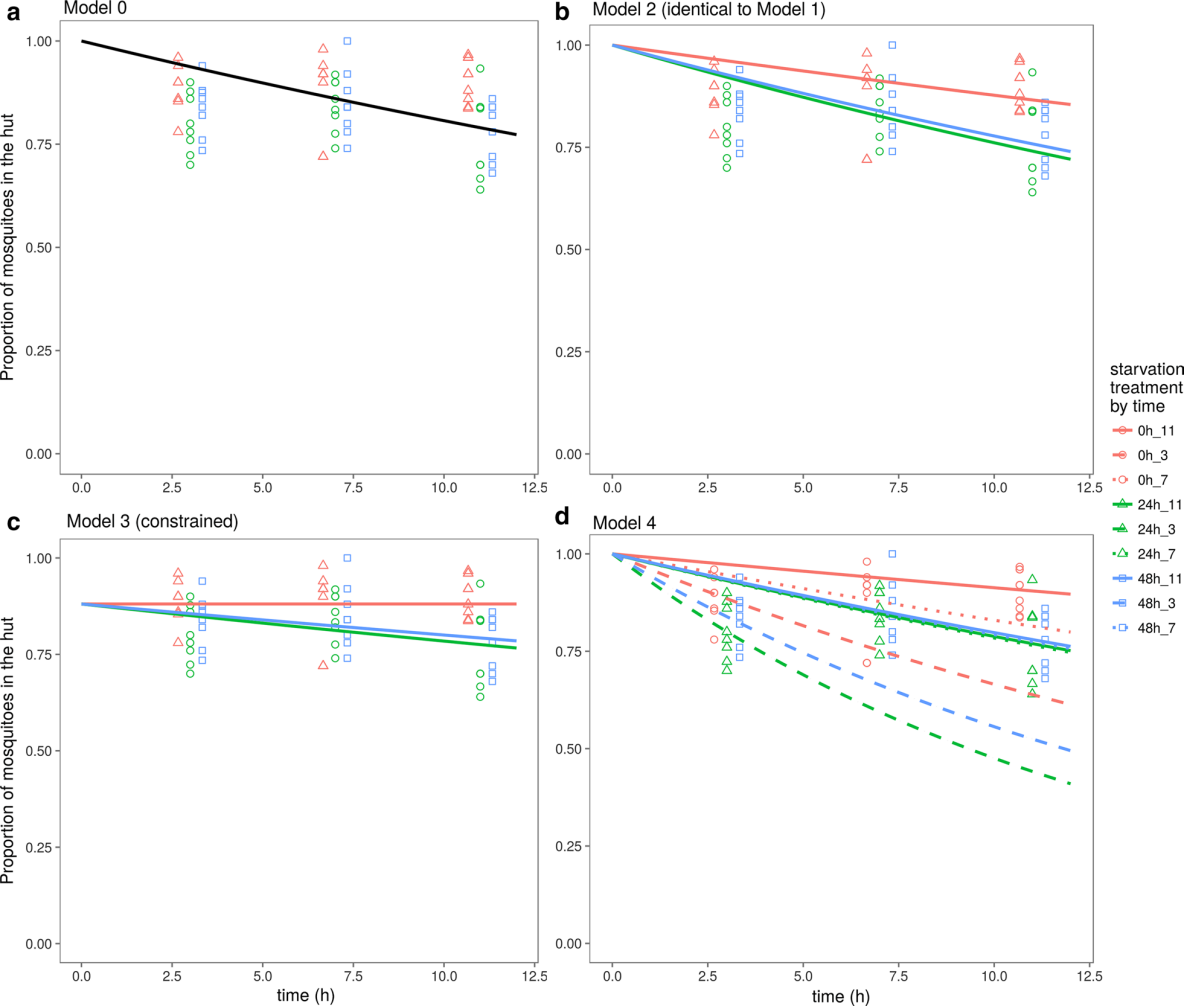
Comparison of fitted models of the proportion of mosquitoes collected in the hut over time. Treatment groups correspond to duration of mosquito starvation treatments (0, 24, 48 h). **a** Model 0, leaving rate is independent of energy state; **b** Model 1 or 2, individuals leave gradually based on energy state; **c** Model 3 (constrained, see text), some proportion of individuals leave immediately, while others leave gradually based on energy state; **d** Model 4, leaving rate depends on both energy state and time

All models fit the experimental data reasonably well, with most parameter estimates significant at the 0.05 level (Table 2) and low residual standard error (Table 3). Plots of the residuals passed visual inspection (see Additional file 2). As a null model against which the other models were compared, Model 0 (Fig. 2) performs worst, having the highest AIC value and highest residual standard error (Table 3), followed by Models 1 and 2, which are mathematically identical. Models 3 and 4 had the lowest AIC scores, and were similar to each other (differing by ~ 2 AIC units), although Model 4 had a lower residual standard deviation than Model 3 (Table 3). Model 3 was also the only model with poor fits for some of the estimated parameters (Table 2). Thus, the best-fitting model, both statistically and conceptually, was Model 4.

**Table 3:** Model performance by AIC scores

Model	AIC	Res. Std. Err	df
0	− 120.3355	0.10275718	1.71
2	− 128.7817	0.09560486	3.69
3a	− 156.9232	0.07811695	4.68
4	− 154.8237	0.07682492	9.63

## Discussion

In recent years, a consensus has emerged that the basic assumption of spatial homogeneity in Ross-MacDonald malaria models is problematic [e.g., 31, 32]. Indeed, data indicates that mosquitoes and their hosts are not well mixed and that directed movement by hosts and vectors could be important factors that determine their spatial distribution [e.g., 33]. Thus, one might wish to describe hosts and vectors as inhabiting discrete patches [34]; however, the spatial resolution required to describe such patches is not clear [35]. This requires an understanding of how and why individual vectors choose to leave one patch and seek others. The current study confirms that such decisions are energy-state dependent.

The foraging of anopheline mosquitoes for energy sources now has both theoretical and empirical support. Further, because such resources feature prominently in their life history exploitation of this behaviour for disease intervention (e.g., toxic baits [5]) makes such tactics much more evolution proof than other tactics (e.g., deployment of broad spread insecticides) [36].

In the experiments described here, the best model that describes mosquito-leaving rates from domiciles is one that includes energy state and time as suggested in “Background” section. As energy state declines in the presence of a protected blood host, individual females are more likely to leave their hut over the course of the evening. In addition, Stone et al. [19] showed that low-energy-state *An. gambiae* females, in the presence of protected blood hosts, were highly likely to seek sugar meals; in nature, that would usually require emigration from the domicile. Caveats from the work presented here include: (i) homogeneous age class tested, (ii) possible bias in recovered versus not-found individuals and (iii) the laboratory strain of mosquitoes may not be representative of wild *An. gambiae* foraging in nature.

Of course, individual mosquitoes might also leave domiciles for reasons separate from nectar foraging. It has been well documented that species such as *An. gambiae* express excito-repellency in the presence of synthetic pyrethroids [37]. It is not clear how energy state and excito-repellency might interact in village settings though one might expect at least an additive effect of danger and energy shortage on leaving rates.

In addition to the evidence for state dependence above, the experiments provide further evidence that *An. gambiae* females actively seek to leave domiciles that harbour protected blood hosts. The estimated leaving rates from Model 4 suggest these leaving rates are non-random, as those mosquitoes with lower energy leave at higher rates whereas those with higher energy levels leave at lower rates. The next question is whether they leave seeking plant hosts. The recent paper by Tenywa et al. [25] shows that females can locate sugar sources within competing vegetation and work at oases show that mosquitoes do find nectar sources [36], but further work is needed to tie together leaving responses per se and sugar exploitation.

In experiments reported here, it is likely that plant odours from nearby fields entered the experimental domiciles, however there is no evidence from Experiment 2 that emigrating females followed these plant-odour plumes to direct their emigration responses There are at least four possible explanations for this discrepancy: (i) the concentration of attractive odours was much higher in the laboratory olfactometer than in the current experiments, making it easier for females to hone in on them; (ii) the air flow was much more laminar in the laboratory olfactometer than in the current experiments, again making it easier for females to hone in on them; (iii) the noise to signal ratio of the sugar odours was much higher in nature than the laboratory, making it difficult for mosquitoes to discriminate between exits; (iv) the *Parthenium hysterophorus* and *Ricinus communis* were simply not attractive under the field It is not possible to discriminate between these explanations without further controlled experiments.

Finally, the authors end by referring back to their attempt to view the results through the lens of simple, underlying mechanisms. Such approaches may provide great utility to help sift through various explanations for outcomes in complex environments.

## Conclusions

These experiments provide some of the first evidence for state-dependent domicile emigration in *An. gambiae*. The results reinforce the notion that *An. gambiae* females make foraging decisions for blood and sugar that are further complicated by bed nets. Finally, it is becoming obvious that such decision making by female mosquitoes is complex and contextual (e.g., [11]), which has implications for interventions in complex environments [19].

## Additional files

**Additional file 1.** Evaluation of mosquito leaving rates based on starvation treatment and experiment end time using logistic regression.

**Additional file 2.** Comparison of fitted models’ residuals of mosquitoes collected in the hut over time. Treatment groups correspond to duration of mosquito starvation treatments (0, 24, 48 hrs). a) Model 0, leaving rate is independent of energy state; b) Model 1 or 2, individuals leave gradually based on energy state; c) Model 3 (constrained, see text), some proportion of individuals leave immediately, while others leave gradually based on energy state; d) Model 4, leaving rate depends on both energy state and time.
