# Supplementary material for: State-dependent domicile leaving rates in Anopheles gambiae
Source: Malar J. 2018 Jan 12;17:25. doi: 10.1186/s12936-017-2166-4 (PMC5767056; doi:10.1186/s12936-017-2166-4)
Supplement: Supplementary file 1 — Additional file 1. Evaluation of mosquito leaving rates based on starvation treatment and experiment end time using logistic regression. [file 12936_2017_2166_MOESM1_ESM.docx]

**Table A1 Evaluation of mosquito leaving rates based on starvation treatment and experiment end time using logistic regression.**

| Parameter | Estimate | Std. Error | z value | Pr(>\|z\|) |
| --- | --- | --- | --- | --- |
| Intercept | -2.245727053 | 0.141370345 | -15.8854183 | < 2.2e-16 |
| Treatment24h | 0.670714911 | 0.119581766 | 5.6088393 | 2.0368804e-08 |
| Treatment48h | 0.546103212 | 0.120352421 | 4.5375341 | 5.6915835e-06 |
| Time | 0.023072375 | 0.014201684 | 1.6246225 | 1.0424303e-01 |
|  |  |  | Null df = 71 | Residual df = 68 |
