# Supplementary figures and images for: State-dependent domicile leaving rates in Anopheles gambiae
Source: Malar J. 2018 Jan 12;17:25. doi: 10.1186/s12936-017-2166-4 (PMC5767056; doi:10.1186/s12936-017-2166-4)

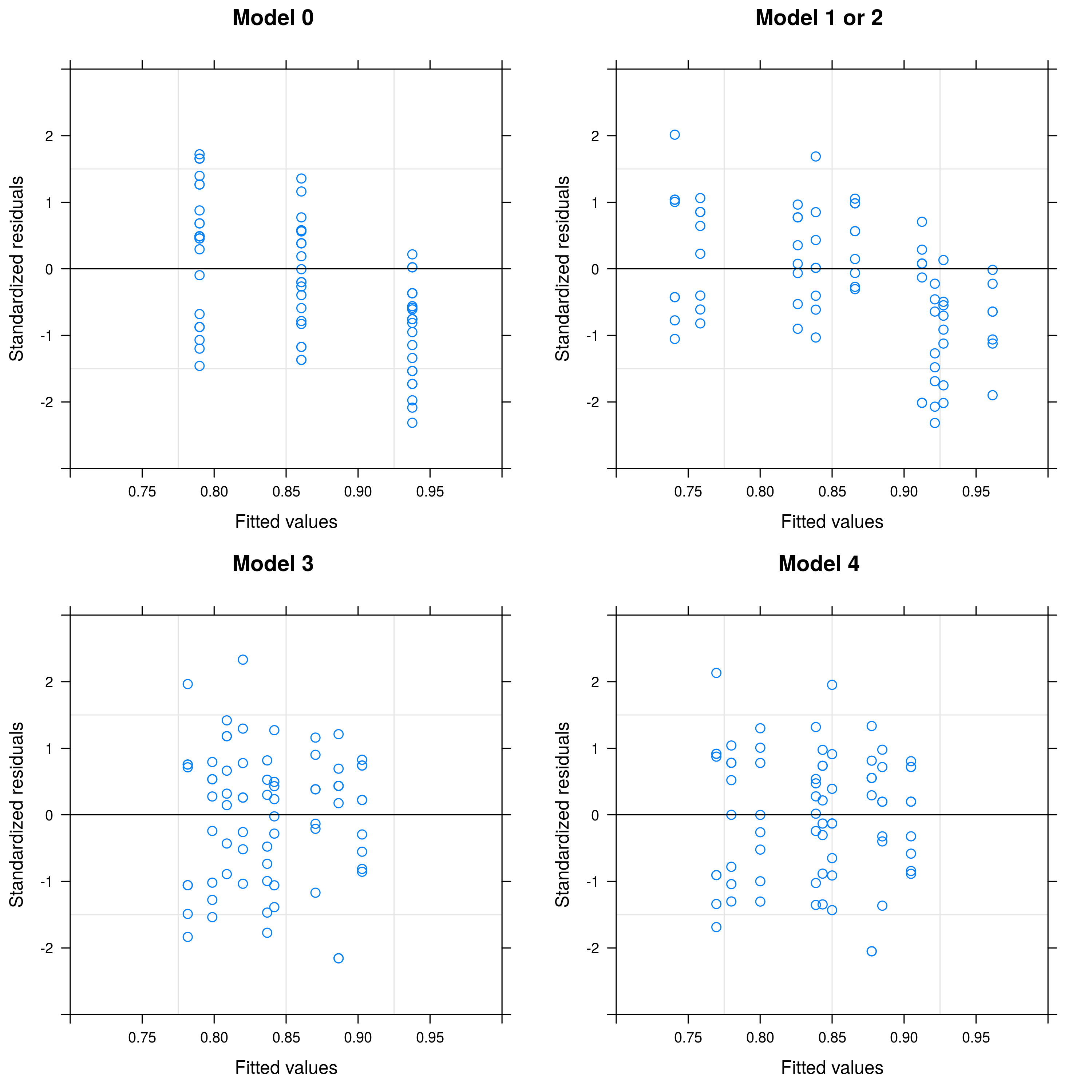

Supplement: Supplementary file 2 — Additional file 2. Comparison of fitted models’ residuals of mosquitoes collected in the hut over time. Treatment groups correspond to duration of mosquito starvation treatments (0, 24, 48 hrs). a) Model 0, leaving rate is independent of energy state; b) Model 1 or 2, individuals leave gradually based on energy state; c) Model 3 (constrained, see text), some proportion of individuals leave immediately, while others leave gradually based on energy state; d) Model 4, leaving rate depends on both energy state and time. [file 12936_2017_2166_MOESM2_ESM.png]
